# Rapid screening and identification of genes involved in bacterial extracellular membrane vesicle production using a curvature-sensing peptide

**DOI:** 10.1128/jb.00497-24

**Published:** 2025-04-04

**Authors:** Hiromu Inoue, Kenichi Kawano, Jun Kawamoto, Takuya Ogawa, Tatsuo Kurihara

**Affiliations:** 1 Institute for Chemical Research, Kyoto University 12918, Uji, Kyoto, Japan; 2 Graduate School of Pharmaceutical Sciences, Kyoto University 12918, Kyoto, Japan; University of Southern California, Los Angeles, California, USA

**Keywords:** extracellular membrane vesicle, vesiculation, *Shewanella vesiculosa*, curvature-sensing peptide

## Abstract

Bacteria secrete extracellular membrane vesicles (EMVs). Physiological functions and biotechnological applications of these lipid nanoparticles have been attracting significant attention. However, the details of the molecular basis of EMV biogenesis have not yet been fully elucidated. In our previous work, an N-terminus-substituted FAAV peptide labeled with nitrobenzoxadiazole (NBD; nFAAV5-NBD) was developed. This peptide can sense the curvature of a lipid bilayer and selectively bind to EMVs even in the presence of cells. Here, we applied nFAAV5-NBD to a genome-wide screening of hyper- and hypo-vesiculation transposon mutants of a Gram-negative bacterium, *Shewanella vesiculosa* HM13, to identify the genes involved in EMV production. We analyzed the transposon insertion sites in hyper- and hypo-vesiculation mutants and identified 16 and six genes, respectively, with a transposon inserted within or near them. Targeted gene-disrupted mutants of the identified genes showed that the lack of putative dipeptidyl carboxypeptidase, glutamate synthase β-subunit, LapG protease, metallohydrolase, RNA polymerase sigma-54 factor, inactive transglutaminase, PepSY domain-containing protein, and Rhs-family protein caused EMV overproduction. On the other hand, disruption of the genes encoding putative phosphoenolpyruvate synthase, d-hexose-6-phosphate epimerase, NAD-specific glutamate dehydrogenase, and sensory box histidine kinase/response regulator decreased EMV production. This study demonstrates the utility of a novel screening method using a curvature-sensing peptide for mutants with altered EMV productivity and provides information on the genes related to EMV production.

**IMPORTANCE:**

Conventional methods for isolation and quantification of extracellular membrane vesicles (EMVs) are generally time-consuming. nFAAV5-NBD can detect EMVs in the culture without separating EMVs from cells. *In situ* detection of EMVs using this peptide facilitated screening of the genes related to EMV production. We succeeded in identifying various genes associated with EMV production of *Shewanella vesiculosa* HM13, which would contribute to the elucidation of bacterial EMV formation mechanisms. Additionally, the hyper-vesiculating mutants obtained in this study would be valuable for EMV applications, such as secreting useful substances as EMV cargoes and producing artificially functionalized EMVs.

## INTRODUCTION

The production of extracellular membrane vesicles (EMVs), nano-sized lipid particles, is commonly observed across all three domains of life (1). Bacteria produce EMVs containing various biomolecules such as proteins, peptidoglycan, phospholipids, and nucleic acids (DNA and RNA) (2). EMVs are involved in diverse biological processes, including horizontal gene transfer, export of cellular metabolites, and intercellular communication (3). In addition to their importance in bacterial physiology, EMVs have potential for various applications, such as serving as a platform for heterologous protein production and as a carrier of biopharmaceuticals (4, 5).

Gram-negative bacteria produce EMVs by outer membrane blebbing and pinching off, as well as by releasing membrane fragments during bacteriolysis (2, 3). It has been documented that membrane blebbing results from a decrease in membrane stability due to disruptions of the crosslinking of the outer membrane and peptidoglycan (6, 7), local curvature formation due to modifications of the asymmetric bilayer of the outer membrane (7–9), and membrane stress caused by the periplasmic accumulation of misfolded proteins, lipopolysaccharides (LPS), and peptidoglycan components (10–13). A variety of vesicle morphologies have been reported, including outer-inner membrane vesicles (14) and multilamellar and multivesicular outer membrane vesicles (15), which are formed by processes different from typical membrane blebbing and cell lysis. Although such insights have been accumulating regarding EMV biogenesis, their molecular basis has not yet been fully clarified, and a comprehensive analysis of the genes related to EMV formation will contribute to elucidating a detailed EMV-producing mechanism.

To investigate genes related to EMV production, the selection of mutants with altered EMV productivity was previously carried out for a gene knockout collection (7) or a random mutation library (6, 16). In these studies, EMV productivity of the mutants was evaluated by immunostaining using an anti-LPS antibody (6, 7) or an antibody against a major cargo protein (16). As far as we know, no study has conducted a rapid evaluation of EMV productivity based on *in situ* detection of EMVs. In our previous research, we designed a curvature-sensing peptide, FAAV, based on a peptide sequence derived from sorting nexin protein 1 (SNX1), which is a Bin/Amphiphysin/Rvs (BAR) family protein (17). Then, by modifying its N-terminus and labeling its C-terminus with a fluorescence group, nitrobenzoxadiazole (NBD), we developed N-terminus-substituted FAAV5 labeled with NBD (nFAAV5-NBD) (18). This peptide can selectively bind to lipid packing defects on EMVs, where the lipids are loosely packed due to high membrane curvature, even in the presence of cells. Therefore, we considered that this peptide could be useful for selecting mutants with altered EMV productivity without removing the cells from the culture, which would significantly increase the throughput of screening.

*Shewanella vesiculosa* HM13, a Gram-negative bacterium isolated from the intestine of a horse mackerel, produces larger amounts of EMVs with less variation in particle size than other well-known *Shewanella* species and *Escherichia coli* (19). The elucidation of the molecular basis behind the EMV production of this strain is expected to provide valuable insights into the mechanisms of bacterial EMV production and morphogenesis. EMVs secreted by this strain harbor a 49 kDa protein, named P49, of unknown function as their single major cargo protein (19, 20). By using P49 as a carrier to transport heterologous proteins to EMVs, this strain could be useful as a host for the production of EMVs with desired properties (19, 21).

In this research, we established a rapid screening method for genes related to EMV production using random transposon mutagenesis and nFAAV5-NBD. The screening resulted in the acquisition of 18 hyper-vesiculating strains and eight hypo-vesiculating strains. We identified the transposon insertion sites in these strains and conducted targeted gene disruption experiments to investigate the involvement of the transposon-disrupted genes in EMV production. Through characterization of EMV production, cell proliferation, and protein secretion of the targeted gene-disrupted strains, we identified eight and four genes whose disruption increased and decreased EMV productivity, respectively. The results suggested that the EMV productivity of *S. vesiculosa* HM13 is regulated by a protein quality control system, extracellular environmental signal sensing, and glutamate metabolism. This study provides a novel screening method using a curvature-sensing peptide and information on genes related to EMV production.

## RESULTS

### Evaluation of EMV-selective binding of nFAAV5-NBD

**Fig 1:**
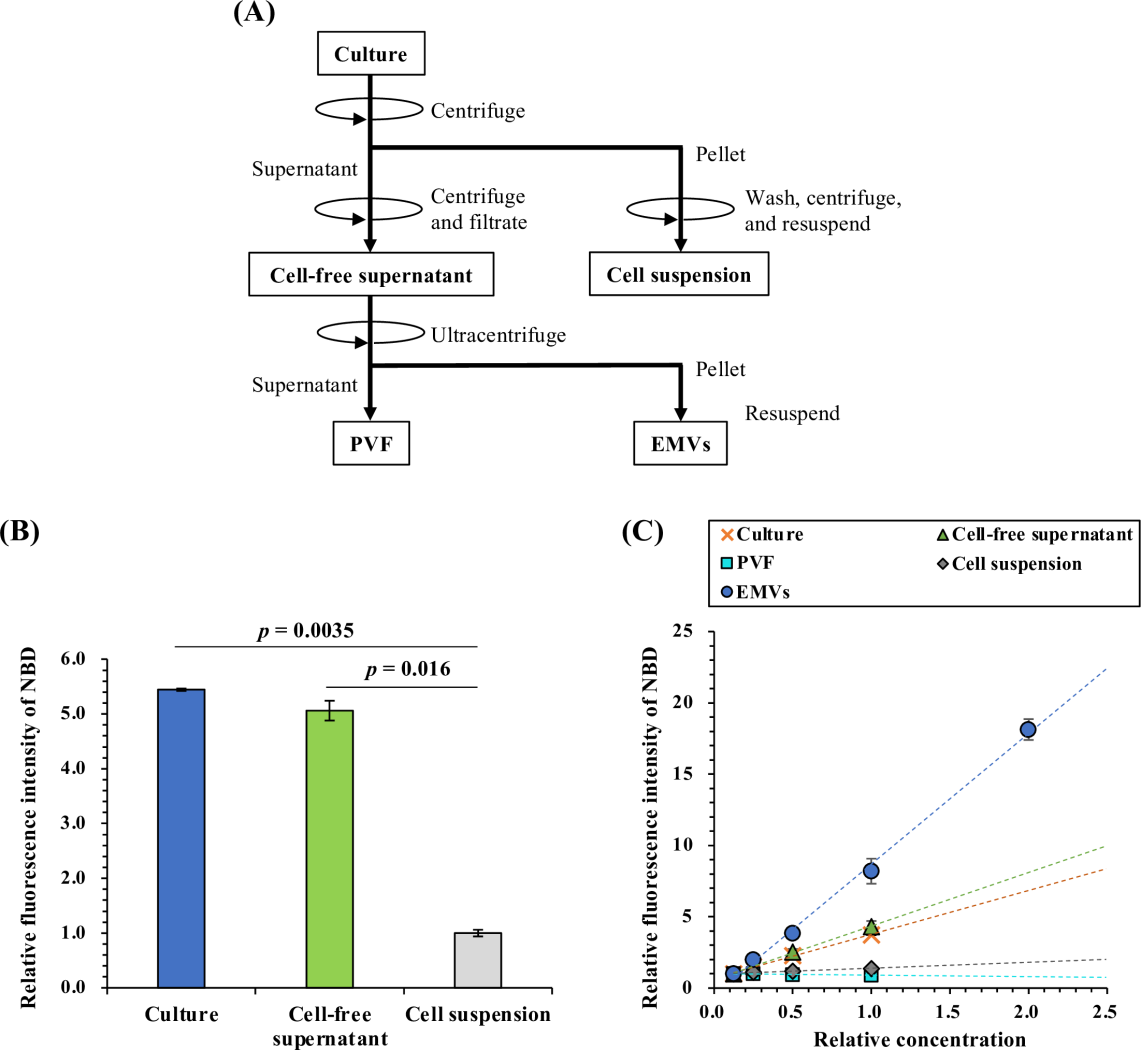
Detection of EMVs in EMV-containing fractions using nFAAV5-NBD. (A) A schematic diagram showing the preparation of the EMV-containing fractions (culture, cell-free supernatant, and EMVs) and the EMV-free fractions (cell suspension and PVF). (B) Detection of EMVs in culture and cell-free supernatant using the curvature-sensing peptide nFAAV5-NBD. Each undiluted fraction (relative concentration of 1.0) was incubated with nFAAV5-NBD. The data are the average ± SD of the values from three independent batches. *P* value (Student’s *t*-test). (C) Measurement of concentration-dependent changes in NBD fluorescence intensity for each fraction. A relative concentration of 1.0 indicates the concentration of each component in the original culture. At a relative concentration of 1.0, the “EMVs” fraction contained 3.0 × 10^10^ particles/mL ± 3.5 × 10^9^ particles/mL (average ± SD) of EMVs as quantified by NTA. The relative fluorescence intensity of each fraction was defined as one at a relative concentration of 0.13. The data are the average ± SD of the values from three independent batches.

To validate whether the NBD-labeled curvature-sensing peptide, nFAAV5-NBD, can detect the EMV concentration in the culture, nFAAV5-NBD was added to the EMV-containing fractions (culture, cell-free supernatant, and EMVs) and the EMV-free fractions (cell suspension and post-vesicle fraction [PVF], which was obtained by removing EMVs from the cell-free supernatant; Fig. 1A). The concentration of each component in the original culture was defined as a relative concentration of 1.0. At this concentration, the “EMVs” fraction contained 3.0 × 10^10^ particles/mL of EMVs as quantified by nanoparticle tracking analysis (NTA). When the fractions without dilution were used, the culture and the cell-free supernatant showed about fivefold higher fluorescence intensity of NBD than the cell suspension (Fig. 1B). The fluorescence intensity of the EMV-containing fractions (culture, cell-free supernatant, and EMVs) increased with increasing relative concentration, whereas such a trend was not observed for the EMV-free fractions (cell suspension and PVF) (Fig. 1C). Thus, this peptide can be applied to the *in-situ* evaluation of EMV productivities of *S. vesiculosa* HM13.

### *In situ* rapid evaluation of EMV productivities of random transposon mutants

**Fig 2:**
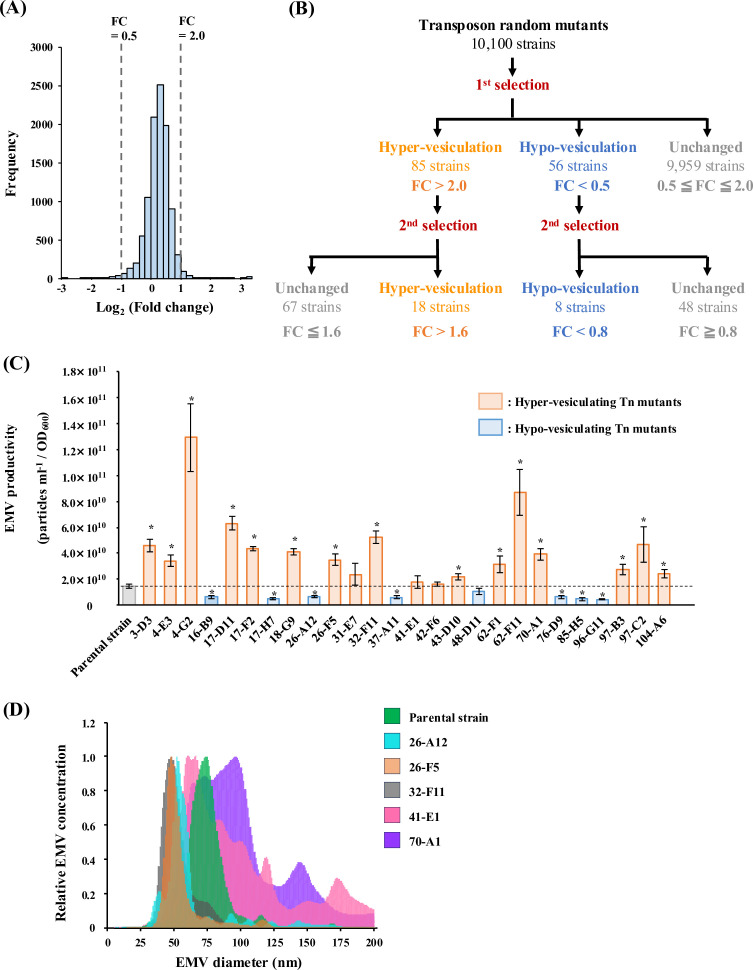
Selection of the Tn mutants using nFAAV5-NBD. (A) Histogram indicating the distribution of fold change (FC) of EMV production in a library of random Tn mutants for the first selection. The mutants with a FC of less than 0.5 and greater than 2.0 (outside the vertical dashed lines) were subjected to the second selection. (B) Flowchart showing the number of subjects and thresholds for each selection. The number of mutants subjected to the first screening was 10,100, which was equivalent to 2.4 times the predicted number of the genes of strain HM13. FC, as defined in “Materials and Methods,” represents the EMV production of the mutants relative to the parental strain quantified using nFAAV5-NBD. (C) EMV productivity of the parental strain and Tn mutants. EMV productivity of the strains was quantified by NTA and normalized to OD_600_. Average ± SE; the parental strain, *n* = 10; Tn mutants, *n* = 3. ∗: *P* < 0.05 (Student’s *t*-test). (D) Hydrodynamic diameter of EMVs of the parental strain and Tn mutants. Particle diameter was quantified by NTA. The parental strain, *n* = 5; Tn mutants, *n* = 3. Each graph shows the average.

The randomness of transposon insertions into the genome was ensured by the identification of transposon insertion sites in 10 randomly selected colonies from the library (data not shown). In the first selection, 10,100 transposon mutants (Tn mutants) were statically cultured in 96-well plates, and their EMV productivities were evaluated by NBD fluorescence intensity and OD value. The base-2 logarithm of the fold change in EMV production by the mutants showed a unimodal broad distribution (average: 0.055, median: 0.068; Fig. 2A), indicating that the EMV productivities of screening-subjected mutants were certainly variable. In the first screening, 85 and 56 mutants were selected as hyper- and hypo-vesiculating candidates, respectively, with fold changes in EMV production greater than 2.0 and less than 0.5 (Fig. 2B). The former and the latter were in the top 0.84% and bottom 0.55%, respectively, of the screening subjects.

To obtain mutants whose EMV productivity increases or decreases regardless of aeration conditions, the 141 candidates selected from the first screening under static culture conditions were aerobically cultivated with shaking and subjected to the evaluation of EMV productivity using nFAAV5-NBD as described in “Materials and Methods.” Eighteen and eight strains were selected as hyper- and hypo-vesiculating strains, respectively, with fold changes in EMV production greater than 1.6 and less than 0.8 (Fig. 2B).

### Characterization of selected Tn mutants

The EMV fractions of the selected Tn mutants were obtained, and the EMV concentration and hydrodynamic diameters were measured by NTA (Fig. 2C and D; Fig. S1A). The results obtained by NTA were consistent with those obtained using nFAAV5-NBD with respect to hyper- and hypo-vesiculation phenotypes for the majority of the mutants (Table S1), although there were four strains whose EMV productions showed no significant differences from that of the parental strain (*P* > 0.05; Fig. 2C). Among 18 mutants selected as hyper-vesiculating strains, 15 mutants produced significantly larger amounts of EMVs compared to the parental strain. Strain 4-G2 exhibited the highest EMV productivity, and its vesiculation level was 9.0-fold higher than that of the parental strain (Fig. 2C). On the other hand, EMV productivity was significantly decreased in the mutants selected as hypo-vesiculating strains except for strain 48-D11, and the lowest EMV productivity was observed for strain 96-G11, which produced 0.31-fold smaller amount of EMVs than the parental strain (Fig. 2C).

The hydrodynamic diameters of EMVs produced by the parental strain and the Tn mutants were compared because screening with nFAAV5-NBD may be influenced not only by the amount of EMVs but also by their size, which could affect their curvature. Analysis of particle size distribution by NTA indicated that the parental strain mainly produced EMVs with a diameter of 60–90 nm (Fig. 2D). Although most strains isolated as hyper- and hypo-vesiculation mutants showed the same particle size distribution as the parent strain (Fig. S1A), the hyper-vesiculating strains 26-F5 and 32-F11 and the hypo-vesiculating strain 26-A12 mainly produced smaller EMVs with a diameter of 40–60 nm than the parental strain (Fig. 2D). The hyper-vesiculating strains 41-E1 and 70-A1 produced slightly larger EMVs than the parental strain with increased size variation (Fig. 2D). Therefore, no correlation between EMV productivity and EMV size was observed for the selected mutants.

Overall, we successfully selected the Tn mutants with increased or decreased EMV productivity using nFAAV5-NBD.

### Identification of transposon insertion sites

Transposon insertion sites were identified by inverse PCR (22) or single-primer PCR (23). Transposon insertion sites identified for 18 hyper-vesiculating strains are listed in Table 1. In brief, transposons were inserted into the coding region of 16 genes and one 5′-untranslated region (UTR). Among the transposon-inserted genes, two genes were predicted to encode inner membrane proteins (HM2192 and HM502) with seven and eight transmembrane helices, respectively, two genes to encode outer membrane proteins (HM1685 and HM2827) not containing predicted transmembrane regions and six genes to encode cytoplasmic proteins (HM3484, HM1858, HM2418, HM3946, HM2929, and HM3068). For the other six proteins (HM4090, HM1880, HM1399, HM3230, HM1963, and HM1528), PSORTb and SOSUI programs did not predict the localization or did not yield consistent results. In strain 26-F5, the transposon was inserted in the 5′-UTR of *hm2720* and *hm2721*, which encode a putative Gate domain-containing spore maturation protein and 16S rRNA methyltransferase RsmF, respectively, disrupting the putative promoter region of *hm2721* and separating the putative promoter of *hm2720* from the coding region (Fig. S2A). In the 41-E1 and 70-A1 strains, the transposon was inserted into different positions of *hm2192*, encoding a putative inactive transglutaminase. By BLASTP search, there were no sequence homologs of HM1880, HM1858, and HM1528 with predictable functions. However, the AlphaFold2 predicted models of HM1880 and HM1528 showed structural similarity to LapG protease from *Legionella pneumophila* (Cα root mean square deviation [RMSD]: 1.8 Å) and an adaptor protein required for acetylation of the alginate exopolysaccharide from *Pseudomonas aeruginosa* (Cα RMSD: 2.1 Å), respectively (Fig. S3). There was no protein with a similar three-dimensional structure to HM1858.

**TABLE 1:** Transposon insertion sites of the hyper-vesiculating strains

Strain	Transposon insertion site	Predicted function of the protein^*c*^	Localization		Accession
			PSORTb^*d*^	SOSUI GramN^*e*^	
3-D3	*hm4090*	Dipeptidyl carboxypeptidase	Not predictable	Outer membrane	LC816094
4-E3	*hm3484*	Glutamate synthase (NADPH) beta subunit	Cytoplasmic	Cytoplasmic	LC816095
4-G2	*hm1880*	LapG protease (4FGO)^*b*^	Not predictable	Cytoplasmic	LC816096
17-D11	*hm1399*	Site-specific integrase	Not predictable	Not predictable	LC816097
17-F2	*hm1858*	Hypothetical protein	Cytoplasmic	Cytoplasmic	LC816098
18-G9	*hm2418*	Metallohydrolase	Cytoplasmic	Cytoplasmic	LC816099
26-F5	*hm2720^a^*	Gate domain-containing spore maturation protein	Inner membrane	Inner membrane	LC816100
26-F5	*hm2721^a^*	16S rRNA methyltransferase RsmF	Cytoplasmic	Cytoplasmic	LC816101
31-E7	*hm1685*	Flagellar L-ring protein FlgH	Outer membrane	Outer membrane	LC816102
32-F11	*hm3946*	RNA polymerase sigma-54 factor RpoN	Cytoplasmic	Cytoplasmic	LC816103
41-E1	*hm2192*	Inactive transglutaminase	Inner membrane	Inner membrane	LC816104
42-F6	*hm502*	PepSY domain-containing protein	Inner membrane	Inner membrane	LC816105
43-D10	*hm3230*	Acyl-CoA dehydrogenase family protein	Cytoplasmic	Outer membrane	LC816106
62-F1	*hm2929*	Superfamily II DNA or RNA helicase	Cytoplasmic	Cytoplasmic	LC816107
62-F11	*hm2827*	Rhs-family protein	Outer membrane	Outer membrane	LC816108
70-A1	*hm2192*	Inactive transglutaminase	Inner membrane	Inner membrane	LC816104
97-B3	*hm1963*	Cupin domain-containing mannose-6-phosphate isomerase	Not predictable	Cytoplasmic	LC816109
97-C2	*hm1528*	Adaptor protein required for acetylation of the alginate exopolysaccharide (6D10)^*b*^	Not predictable	Periplasmic	LC816110
104-A6	*hm3068*	DNA-binding ATP-dependent protease La	Cytoplasmic	Cytoplasmic	LC816111

^*a*^ Transposon was inserted in the 5′-UTR of the corresponding genes.

^*b*^ A protein with similar tertiary structure (PDB No.) as shown in Fig. S2.

^*c*^ The function was predicted based on homology searches using BLASTP (https://blast.ncbi.nlm.nih.gov/Blast.cgi?PAGE=Proteins).

^*d*^ https://www.psort.org/psortb/.

^*e*^ https://harrier.nagahama-i-bio.ac.jp/sosui/sosuigramn/sosuigramn_submit.html.

On the other hand, in eight hypo-vesiculating strains, transposons were inserted into six genes and one 5′-UTR (Table 2). Among the genes with transposon insertions, one gene was predicted to encode inner membrane protein (HM3454) containing seven transmembrane helices, and two genes were predicted to encode cytoplasmic proteins (HM2766 and HM369). For the other proteins (HM2775, HM2704, and HM3986), PSORTb and SOSUI programs did not yield consistent results. In the 37-A11 strain, the transposon was inserted into the region between the putative promoter of *hm239*, encoding 23S rRNA methylase RlmKI, and its coding region, probably without affecting the expression of *hm238* (Fig. S2B). The function of HM238, a short peptide chain consisting of 39 amino acid residues, could not be predicted based on its sequence and predicted structure. In the 76-D9 and 96-G11 strains, the transposon was inserted into different sites of *hm2704*, encoding putative NAD-specific glutamate dehydrogenase (GDH).

**TABLE 2:** Transposon insertion sites of the hypo-vesiculating strains

Strain	Transposon insertion site	Predicted function of the protein^*b*^	Localization		Accession
			PSORTb^*c*^	SOSUI GramN^*d*^	
16-B9	*hm2766*	Phosphoenolpyruvate synthase	Cytoplasmic	Cytoplasmic	LC816112
17-H7	*hm2775*	d-Hexose-6-phosphate epimerase	Not predictable	Extracellular	LC816113
26-A12	*hm369*	Molybdopterin molybdotransferase MoeA	Cytoplasmic	Cytoplasmic	LC816114
37-A11	*hm238^a^*	Hypothetical protein	Inner membrane	Not predictable	LC816115
37-A11	*hm239^a^*	23S rRNA methylase RlmKI	Cytoplasmic	Cytoplasmic	LC816116
48-D11	*hm3454*	CcsA-related protein	Inner membrane	Inner membrane	LC816117
76-D9	*hm2704*	NAD-specific GDH	Not predictable	Cytoplasmic	LC816118
85-H5	*hm3986* *90*	Sensory box histidine kinase/response regulator	Inner membrane	Cytoplasmic	LC816119
96-G11	*hm2704*	NAD-specific GDH	Not predictable	Cytoplasmic	LC816118

^*a*^ Transposon was inserted in the 5′-UTR of the corresponding genes.

^*b*^ The function was predicted based on homology searches using BLASTP (https://blast.ncbi.nlm.nih.gov/Blast.cgi?PAGE=Proteins).

^*c*^ https://www.psort.org/psortb/.

^*d*^ https://harrier.nagahama-i-bio.ac.jp/sosui/sosuigramn/sosuigramn_submit.html.

### Verification of changes in EMV production by targeted gene disruption and complementation

**Fig 3:**
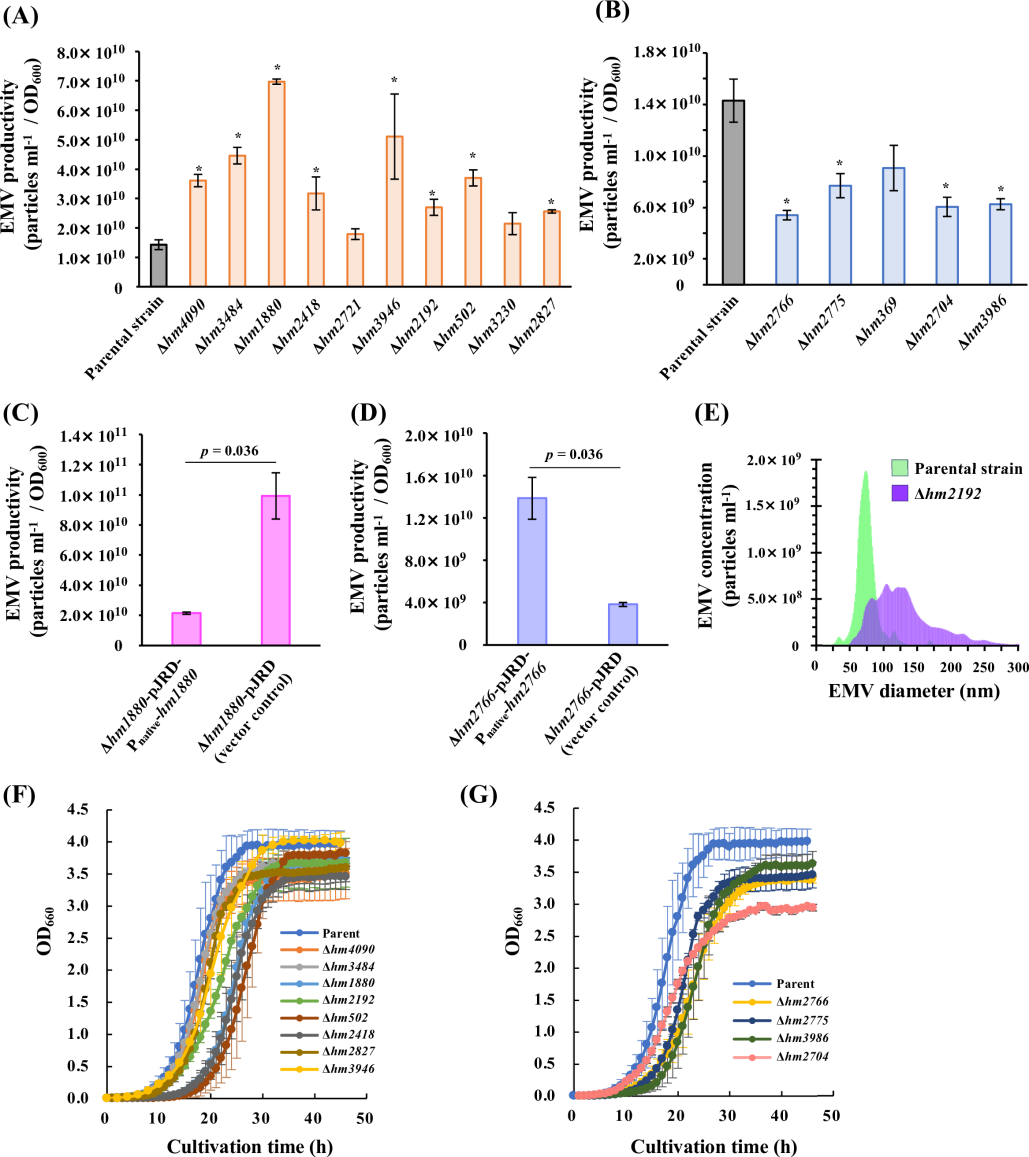
Characteristics of EMV production and growth of the gene-disrupted strains. (A and B) EMV productivity of the parental strain and the targeted gene-disrupted strains (A: hyper-vesiculating mutants and B: hypo-vesiculating mutants). EMV concentration was quantified by NTA and normalized to OD_600_. Average ± SE; the parental strain, *n* = 10; the mutants, *n* = 3. ∗: *P* < 0.05 (Student’s *t*-test). (C and D) EMV productivity of the complemented strains (C: *hm1880* complementation and D: *hm2766* complementation). EMV production was quantified by NTA and normalized to OD_600_. Average ± SE; n = 3. Student’s *t*-test. (E) Hydrodynamic diameter of EMVs of the parental strain and *hm2192*-disrupted strain (Δ*hm2192*). Particle diameter was quantified by NTA. The parental strain, *n* = 5; Δ*hm2192*, *n* = 3. Each graph shows the average. (F and G) Growth curve of the parental strain and the targeted gene-disrupted strains (F: hyper-vesiculating mutants and G: hypo-vesiculating mutants). The growth of the strains was evaluated by monitoring OD_660_ with BioPhoto recorder TVS062CA. The parental strain, *n* = 5; the mutants, *n* = 3.

To examine whether the identified genes are involved in EMV production by *S. vesiculosa* HM13, we generated the targeted gene-disrupted mutants by single homologous recombination using a knock-out plasmid, pKNOCK-Km (24). The genes that do not have enough length for single homologous recombination were not disrupted in this study. Quantification of EMVs by NTA revealed that Δ*hm4090*, Δ*hm3484*, Δ*hm1880*, Δ*hm2418*, Δ*hm3946*, Δ*hm2192*, Δ*hm502*, and Δ*hm2827* had significantly higher EMV productivity, and their EMV productions were 2.5-, 3.1-, 4.9-, 2.2-, 3.6-, 1.9-, 2.6-, and 1.8-fold higher than that of the parental strain, respectively (Fig. 3A). Δ*hm2766*, Δ*hm2775*, Δ*hm2704*, and Δ*hm3986* exhibited 0.38-, 0.54-, 0.42-, and 0.44-fold lower EMV productions than the parental strain with significant differences, respectively (Fig. 3B). The results suggest that the genes disrupted in these strains are involved in EMV production by strain HM13. Complementation experiments were performed for Δ*hm1880* and Δ*hm2766*, which showed the most significant increase and decrease in EMV production, respectively, to confirm that the changes in EMV production in these mutants were caused by the disruption of *hm1880* and *hm2766*, respectively. The *hm1880*-complemented strain and the *hm2766*-complemented strain produced 0.22-fold fewer and 3.6-fold more EMVs, respectively, compared to the strains carrying the vector control (Fig. 3C and D), and their EMV production levels were comparable to that of the parental strain (Fig. 3A through D).

The hyper-vesiculating strain Δ*hm2192* produced EMVs with a large variation in size and mainly produced EMVs with a larger diameter of 75–125 nm than the parental strain (Fig. 3E). This morphological characteristic was consistent with that observed for the Tn mutants (41-E1 and 70-A1; Fig. 2D). This result suggests that HM2192 is related to the EMV particle size regulation in strain HM13. The other hyper-vesiculating strains (Fig. S1B) and hypo-vesiculating strains (Fig. S1C) mainly produced EMVs with size distributions almost identical to that of the parental strain.

### Growth characteristics of the targeted gene-disrupted strains

The growth of the parental strain and the targeted gene-disrupted strains was monitored (Fig. 3F and G). Δ*hm1880*, Δ*hm2418*, Δ*hm502*, Δ*hm2766*, Δ*hm2775*, and *Δhm3986* showed longer lag phases than the parental strain. The doubling time of the parental strain was 2.2 h. Δ*hm2192* and Δ*hm2704* required longer proliferation times than the parental strain, with a doubling time of approximately 3.0–3.5 h. Although the defects in several identified genes caused prolonged lag phase and changes in doubling time and cell yields, these mutants did not exhibit cell aggregation and lysis until the early stationary phase under the tested conditions.

### Secretory protein production of the targeted gene-disrupted strains

**Fig 4:**
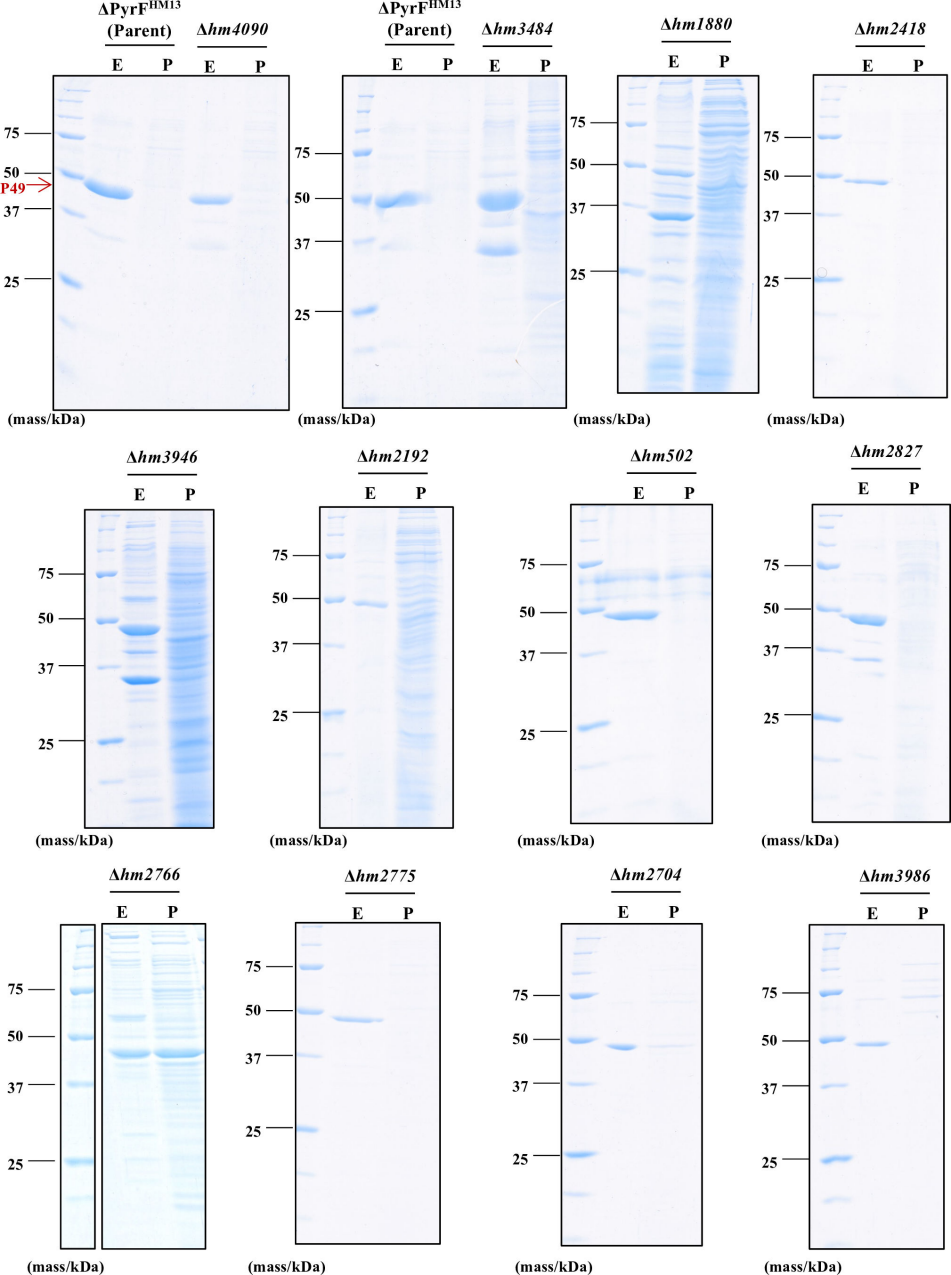
Analysis of the secretory proteins from the parental strain and the targeted gene-disrupted strains. EMVs and PVF from 300 µL of culture were subjected to trichloroacetic acid precipitation and SDS-PAGE. The gel was stained with Coomassie Brilliant Blue G-250. The band with an arrow was identified as P49 by western blotting with an anti-P49 antibody. E: EMVs and P: PVF.

The EMV fractions and PVFs collected from the parental strain and the targeted gene-disrupted strains were subjected to SDS-PAGE to analyze secretory protein productions (Fig. 4). As reported in our previous research (19, 20), a single major cargo protein P49 was observed in the EMV fraction collected from the parental strain with an apparent molecular mass of about 49 kDa. Similar to the parental strain, the major protein band in the EMV fractions from the mutants was identified as P49 by western blot analysis using an anti-P49 antibody (data not shown). However, many other protein bands were also observed in EMV fractions and PVFs from Δ*hm3484*, Δ*hm1880*, Δ*hm3946,* Δ*hm2192*, and Δ*hm2766*, indicating that these mutants secreted numerous proteins, other than P49, that were contained or not contained in EMVs.

## DISCUSSION

### Summary of *in situ* rapid screening using a curvature-sensing peptide

**Fig 5:**
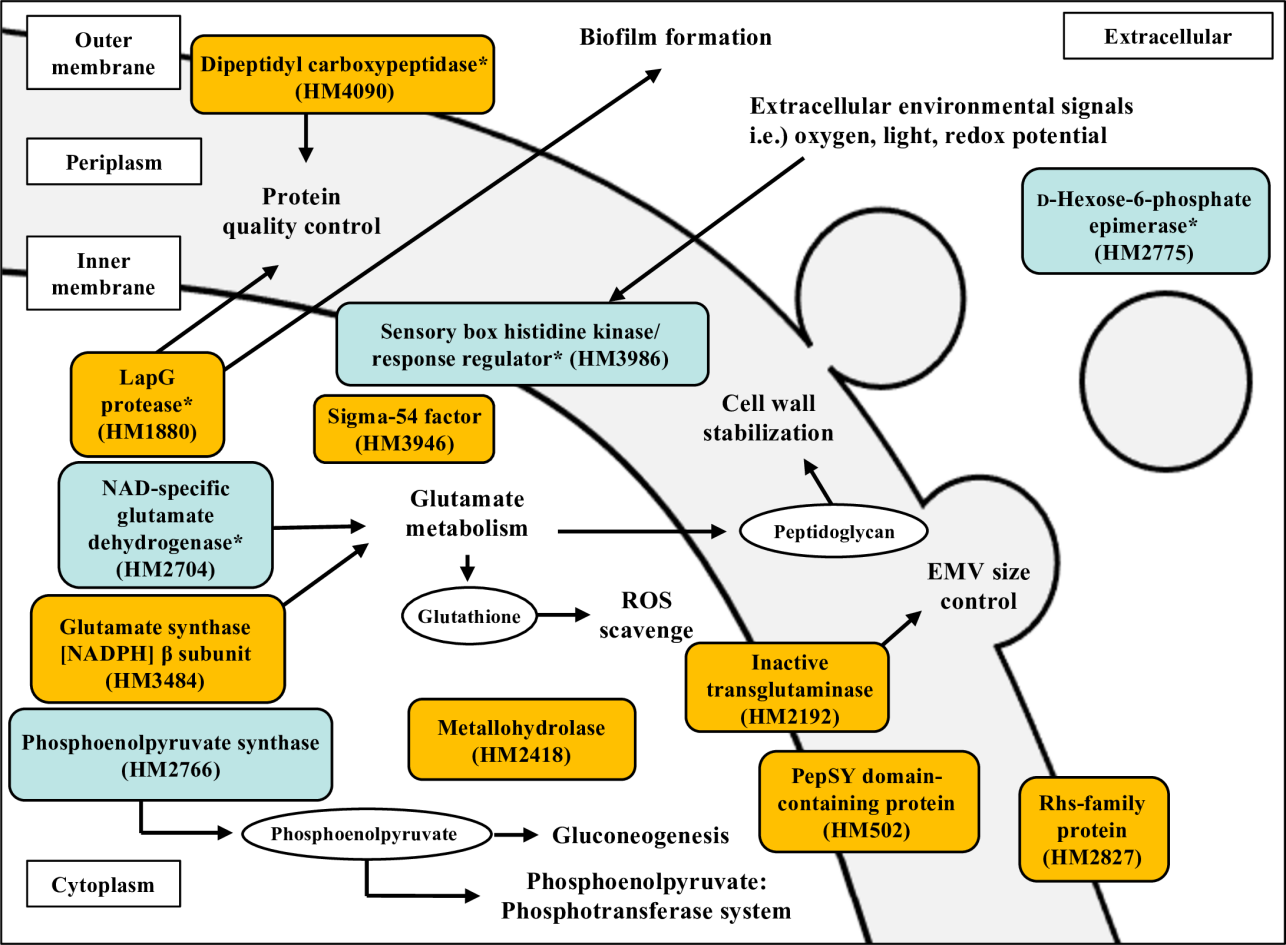
Proteins suggested to be involved in EMV production by *S. vesiculosa* HM13. Predicted localization and functions of the proteins encoded by the genes whose disruptions increased (orange boxes) or decreased (blue boxes) EMV productivity are shown. Localizations were predicted by the PSORTb and SOSUI programs. Proteins with low localization reliability are marked with *.

The physiology and biotechnological applications of EMVs have been attracting significant attention (2, 3). However, the mechanism of bacterial EMV production has not been fully understood, and many unidentified genes are supposed to be involved in this process (25). In this work, to identify the genes involved in EMV biogenesis, we established a novel high-throughput screening method for Tn mutant library with a curvature-sensing peptide, nFAAV5-NBD (18). The results of the assay for EMVs produced by *S. vesiculosa* HM13 indicated that nFAAV5-NBD could be applied to *in situ* selection of the strains with variable EMV productivity without separation of cells and EMVs (Fig. 1). By evaluating the amount of EMVs *in situ*, we succeeded in selecting mutants with altered EMV production (Fig. 2), and the genes related to EMV production by strain HM13 were identified (Fig. 5).

For genome-wide assessment, we subjected 10,100 mutants to the present experiment, corresponding to approximately 2.4 times the estimated number of the genes in strain HM13. Some of the genes were identified for more than one mutant in the screening (Tables 1 and 2). In the previous study, Kulp et al. conducted a high-throughput selection of hyper- and hypo-vesiculating strains from the whole-genome knockout library of *E. coli* mutant strains (Keio collection) (7). They elucidated that the knockout of the genes responsible for LPS and enterobacterial common antigen leads to hyper-vesiculation, whereas the knockout of the genes functioning in oxidative stress response pathways leads to hypo-vesiculation. However, the homologs of these genes were not identified in our present study. The present screening employed static culture in 96-well plates for the first selection of mutants (Fig. 2B), while in the screening conducted by Kulp et al., shaking culture was used (7), suggesting that these differences in culture conditions caused differences in the identified genes. It is also possible that there are bacterial species-specific factors affecting EMV production, or the Tn mutant library used in this study did not contain the mutants with disruptions in the genes identified in the previous study.

By nFAAV5-based screening, we selected 18 hyper-vesiculating mutants and 8 hypo-vesiculating mutants. We succeeded in identifying 22 genes and two 5′-UTRs where transposons were inserted (Tables 1 and 2). Notably, we elucidated the involvement of various proteins predicted to be localized at the outer membrane (HM1685 and HM2827) and the inner membrane (HM2192, HM502, and HM3454). Interestingly, disruption of *hm2192*, annotated as the gene encoding an inactive transglutaminase fused to seven transmembrane helices (Table S2), affected not only EMV production but also EMV size uniformity (Fig. 3A and E). Amidoligases, including transglutaminase, were reported to be involved in amide modifications to polysaccharides and proteins (26), but their function in EMV production remains to be elucidated.

### Enhancement of EMV production by defects in proteins involved in protein quality control

The gene encoding a homolog of dipeptidyl carboxypeptidase (Dcp; HM4090) was identified as the transposon-disrupted gene in one of the hyper-vesiculating mutants (Table 1). Dcp is the protease that removes dipeptides from the C-termini of N-blocked tripeptides, tetrapeptides, and larger peptides (27). HM4090 was predicted to be an outer membrane-localized lipoprotein with an N-terminal lipid-binding site by SOSUI and SignalP 6.0 programs and to play a role in protein quality control in the outer membrane or periplasm. Membrane stress caused by an accumulation of misfolded proteins is one of the most well-known vesiculation mechanisms (2, 28). Previously, it was shown that the deletion of DegP, a periplasmic protease/chaperone, increased EMV production by *E. coli* (28). Therefore, membrane stress resulting from protein misfolding would cause hyper-vesiculation in Δ*hm4090*.

Δ*hm1880* exhibited a 4.9-fold increase in EMV production compared to the parental strain, the highest EMV productivity observed in this study (Fig. 3A). Structure prediction by AlphaFold2 revealed that HM1880 has a LapG protease-like fold (Fig. S3A and B). LapG is a periplasmic protease constituting the LapADG system, which is responsible for periplasmic proteolysis controlled by c-di-GMP-mediated signaling (29). Disruption of *hm1880* may cause accumulation of misfolded proteins in the periplasm and increase EMV production, similar to the case of *hm4090* disruption. Verifying this hypothesis, such as identifying proteins that accumulate in the periplasm of these mutants, remains a future task.

### Regulation of EMV production by signal transduction protein

We found that disruption of *hm3986* reduced EMV productivity (Fig. 3B). HM3986, a sensory box histidine kinase/response regulator, contains a sensory box PAS domain and signal transduction domain BaeS (Table S2). PAS domains are conserved in three domains of life and recognize multiple stimuli such as light, oxygen, and redox potential (30). Thus, strain HM13 is supposed to have a PAS and BaeS domain-mediated system for sensing extracellular environmental signals, which is involved in EMV production. Detailed analyses, such as transcriptomic and proteomic analyses of the *hm3986*-disrupted strain, are necessary to understand how HM3986 regulates EMV production. Comparing the EMV productivity of Δ*hm3986* and the parental strain in the presence of redox agents would help understand the redox sensing by HM3986.

### Involvement of metabolic enzymes in the EMV production

We revealed that disruption of two genes that are presumably responsible for glutamate metabolism, *hm3484* and *hm2704*, results in an increase and decrease of EMV production by strain HM13, respectively (Fig. 3A and B). Glutamate metabolism is strictly regulated by the glutamine synthetase-glutamate-oxoglutarate amidotransferase and the GDH cycles that comprise glutamate synthase and glutamate dehydrogenase (31). Disruption of the genes coding for a glutamate synthase β-subunit homolog, HM3484, or a glutamate dehydrogenase homolog, HM2704, would affect the cellular glutamate level. Glutamate is a major precursor of glutathione (32) and peptidoglycan (33), functioning in the scavenging of reactive oxidative species (ROS) and in membrane stability, respectively. Abundant ROS in the cell generates membrane stress and induces EMV production (34). These results suggest that EMV production by *S. vesiulosa* HM13 is regulated by the intracellular glutamate levels and its derivatives.

### Conclusions

In this study, we established the screening method using a curvature-sensing peptide, nFAAV5-NBD, and successfully identified the genes related to EMV production by *S. vesiculosa* HM13 (Fig. 5). The findings indicated that protein quality control, extracellular environmental signal sensing, and glutamate metabolism are involved in EMV production. The targeted gene-disrupted mutants of Dcp (Δ*hm4090*), glutamate synthase (Δ*hm3484*), and Rhs-family protein (Δ*hm2827*) produced significantly larger amounts of EMVs than the parental strains (Fig. 3A) without growth defects (Fig. 3F). These strains harbored a major cargo protein P49 on their EMVs (Fig. 4). These strains are expected to be useful to produce heterologous proteins as cargoes of EMVs by using P49 as a carrier. Further research is required to verify whether curvature-sensing peptide-based screening can be applied to other bacterial species and to elucidate in detail the functions of the genes identified in this study in EMV biogenesis.

## MATERIALS AND METHODS

### Bacterial strains and culture conditions

The strains used in this study are listed in Table S3. A rifampicin (Rif)-resistant mutant of *S. vesiculosa* HM13 with the orotidine-5′-monophosphate decarboxylase (PyrF) gene deleted (∆PyrF^HM13^) was used as the parent strain (35). *E. coli* S17-1/λ*pir* was used as the donor strain in the conjugal gene transfer (36). Gene disruption and random transposon mutagenesis were performed with a knock-out plasmid, pKNOCK-Km (24), and a transposon-containing plasmid, pMiniHimar RB1 (37), respectively (Table S3). Complementation experiments were conducted for ∆*hm1880* and ∆*hm2766* with a broad-host-range vector, pJRD-Cm^r^ (38), carrying *hm1880* and *hm2766*, respectively (Table S3). ∆PyrF^HM13^ and its derivatives were grown at 18°C in Luria-Bertani (LB) medium (1% of tryptone [Nacalai Tesque, Kyoto, Japan], 0.5% of yeast extract [Difco, Becton Dickinson, Franklin Lakes, NJ, USA], and 1% of NaCl [pH 7.0]). *E. coli* S17-1/λ*pir* was cultivated at 37°C in an LB medium. When necessary, supplements were added to the medium in the following concentrations: 50 µg/mL Rif, 50 µg/mL kanamycin (Km), 30 µg/mL chloramphenicol (Cm), and 50 µg/mL uracil (Ura). The cultivation was performed in BioShaker BR-43FL (Taitec, Saitama, Japan) at 180 rpm and evaluated with spectrophotometer UV-2450 (Shimadzu, Kyoto, Japan) by monitoring an optical density at 600 nm (OD_600_). The growth of ∆PyrF^HM13^ and its derivatives was evaluated by monitoring OD_660_ at 70 rpm and 18°C (BioPhoto recorder TVS062CA, ADVANTEC Toyo, Tokyo, Japan).

### Isolation of EMVs by ultracentrifugation

∆PyrF^HM13^ and its derivatives were grown in 5 mL of LB medium and harvested at the early stationary phase (OD_600_ of about 2.0). The culture was centrifuged at 6,800 × *g* for 10 min at 4°C. The cells were washed with Dulbecco’s phosphate-buffered saline (DPBS) (39) and resuspended in DPBS. The cell suspension was stored until use for the evaluation of nFAAV5-NBD-binding specificity. The supernatant was centrifuged at 13,000 × *g* for 15 min at 4°C to remove the remaining bacterial cells. The supernatant was filtered through a 0.45 µm pore polyethersulfone filter to remove the remaining debris, and the filtrate was collected as a cell-free supernatant fraction. The cell-free supernatant fraction was stored until use for the evaluation of nFAAV5-NBD-binding specificity. EMVs were obtained from 3 mL of filtrate by ultracentrifugation at 100,000 × *g* (average centrifugal force) and 4°C for 2 h with 50Ti rotor and Optimax centrifuge (Beckman Coulter, Brea, CA, USA). The supernatant after removing EMVs was collected as PVF. The pellets of EMVs were resuspended in 300 µL of DPBS and collected as an EMV fraction. PVF and the EMV fraction were used for the evaluation of nFAAV5-NBD-binding specificity and the analysis of secretory proteins by SDS-PAGE and Coomassie Brilliant Blue G-250 (CBB) staining.

### Evaluation of nFAAV5-NBD-binding specificity

The EMV-containing fractions (culture, cell-free supernatant, and EMVs) and the EMV-free fractions (cell suspension and PVF) were diluted with LB medium (for culture, cell-free supernatant, and PVF) or DPBS (for EMVs and cell suspension). The concentration of each component in the original culture was defined as a relative concentration of 1.0. Each fraction (100 µL) was transferred to 96-well black flat bottom plates (Greiner, Frickenhausen, Germany), and then nFAAV5-NBD was added at a final concentration of 0.5 µM. After 5 min of incubation at room temperature, the fluorescence intensity of NBD was measured with Infinite 200 Pro M Nano+ (Tecan, Männedorf, Switzerland) at 480 nm excitation and 530 nm emission. The data were obtained under optimized experimental conditions (e.g., gain: 100 and the *z*-position of the luminescence fiber bundle: 20,000 µm).

### Random transposon mutagenesis and screening using nFAAV5-NBD

The random transposon mutagenesis was conducted by conjugation of ∆PyrF^HM13^ and *E. coli* S17-1/λ*pir* harboring pMiniHimar RB1. Single colonies of Tn mutants formed on LB agar plates containing Rif, Km, and Ura were isolated and inoculated into 150 µL of LB liquid medium supplemented with Ura in 96-well culture plates. Following static culture for 24 h at 18°C, all Tn mutants were subcultured in 150 µL of fresh LB media (1: 20 dilution rate) containing Ura and statically cultivated at 18°C for 24 h, when the OD_600_ of ΔPyrF^HM13^ reached about 1.8 (Fig. S4). After cultivation, 100 µL culture was transferred into 96-well black flat bottom plates (Greiner), and nFAAV5-NBD was added to each well at a final concentration of 0.5 µM. After 5 min of incubation at room temperature, the fluorescence intensity of NBD was measured with Infinite 200 Pro M Nano+ (Tecan) at 480 nm excitation and 530 nm emission. Fold change of EMV production was defined as follows: Fold change = (NBD fluorescence intensity/OD_600_)_Tn_/(NBD fluorescence intensity/OD_600_)_parent_ (“Tn” and “parent” indicate the values of the Tn mutant culture and the parental strain culture, respectively). The strains showing greater than 2.0- or less than 0.5-fold EMV production were selected as hyper- or hypo-vesiculating candidates, respectively (first selection). The selected candidates were aerobically cultured in 5 mL of LB medium containing Ura (18°C, 180 rpm [BioShaker BR-43FL, Taitec], 24 h), and EMV production was again evaluated using nFAAV5-NBD. Candidates that finally showed fold change greater than 1.6 and less than 0.8 were selected as hyper- and hypo-vesiculating strains, respectively (second selection).

### Identification of transposon insertion sites

To identify the insertion sites of the transposon, we performed rapid amplification of the regions adjacent to the transposon ends using a single primer (Table S4; Single-Primer-PCR-1, Single-Primer-PCR-2, or Single-Primer-PCR-3). All the Tn mutants were subjected to single-primer PCR (23), and the PCR products were sequenced with a nested primer (Table S4; Single-Primer-PCR-nested), resulting in the identification of the transposon insertion sites of the Tn mutants except for strains 3-D3 and 18-G9. The genomic DNA extracts of strains 3-D3 and 18-G9 were digested with the restriction enzymes, XbaI (Takara Bio, Shiga, Japan) and SacI (Takara Bio), and circularized DNA prepared by self-ligation with Ligation High Ver.2 (Toyobo, Osaka, Japan) was used as a template for inverse PCR (22). The genomic DNA region flanked by the two ends of the transposon in the circular DNA was amplified and sequenced by two primers (Table S4; Himar1 and Himar615), which annealed specifically to different strands of the transposon. The sequences of the proteins encoded by the identified genes were subjected to the BLASTP (https://blast.ncbi.nlm.nih.gov/Blast.cgi?PAGE=Proteins) analysis. Proteins in the database with sequence similarity to the subject proteins are listed in Tables 1 and 2, and the conserved domains found in the subject proteins are listed in Table S2. For HM1880 and HM1528, no homologous proteins or conserved domains were found. To predict their functions, their sequences were submitted to AlphaFold2 version 1.4 (https://colab.research.google.com/github/sokrypton/ColabFold/blob/main/AlphaFold2.ipynb) to generate conformational models. Based on these models, we searched for structurally similar proteins in the Protein Data Bank (https://www.rcsb.org/) using the Dali server (http://ekhidna.biocenter.helsinki.fi/dali/). Protein localization was predicted by PSORTb (https://www.psort.org/psortb/) and SOSUI programs (https://harrier.nagahama-i-bio.ac.jp/sosui/sosuigramn/sosuigramn_submit.html). Signal peptides were predicted by SignalP 6.0 (https://services.healthtech.dtu.dk/services/SignalP-6.0/).

### Targeted gene disruption by single crossover recombination

A linear fragment of pKNOCK-Km was amplified with the primers pKNOCK-1 and -2 (Table S4). The internal fragments of each gene to be disrupted were amplified using the primers specific to the target genes (Table S4; hm4090-single-FW and -RV for *hm4090* disruption; hm3484-single-FW and -RV for *hm3484* disruption; hm1880-single-FW and -RV for *hm1880* disruption; hm2418-single-FW and -RV for *hm2418* disruption; hm2721-single-FW and -RV for *hm2721* disruption; hm3946-single-FW and -RV for *hm3946* disruption; hm2192-single-FW and -RV for *hm2192* disruption; hm502-single-FW and -RV for *hm502* disruption; hm3230-single-FW and -RV for *hm3230* disruption; hm2827-single-FW and -RV for *hm2827* disruption; hm2766-single-FW and -RV for *hm2766* disruption; hm2775-single-FW and -RV for *hm2775* disruption; hm369-single-FW and -RV for *hm369* disruption; hm2704-single-FW and -RV for *hm2704* disruption; and hm3986-single-FW and -RV for *hm3986* disruption). The amplified linear fragment of pKNOCK-Km and the internal fragments of each gene were fused using the In-Fusion HD Cloning Kit (Takara Bio) according to the manufacturer’s instructions. The insertion of the internal fragments into an appropriate site on pKNOCK-Km was confirmed by PCR with a primer pair of pKNOCK-check-FW and pKNOCK-check-RV (Table S4). ∆PyrF^HM13^ was conjugated with *E. coli* S17-1/λ*pir* transformed with the plasmids thus constructed, and then the transconjugants were selected on 1.5% LB agar plates containing Rif, Km, and Ura. The insertion of plasmids into an appropriate site on the genome was confirmed by PCR with a primer pair of pKNOCK-check-RV and one of the following primers: hm4090-check-FW, hm3484-check-FW, hm1880-check-FW, hm2418-check-FW, hm2721-check-FW, hm3946-check-FW, hm2192-check-FW, hm502-check-FW, hm3230-check-FW, hm2827-check-FW, hm2766-check-FW, hm2775-check-FW, hm369-check-FW, hm2704-check-FW, and hm3986-check-FW (Table S4).

### Complementation experiments for gene-disrupted strains

A linear fragment of pJRD-Cm^r^ was amplified with the primers pJRD-Cm^r^-1 and −2 (Table S4). The coding regions of *hm1880* and *hm2766* together with their 812 and 542 bp upstream regions, respectively, including the putative promoters predicted by Berkeley Drosophila Genome Project Neural Network Promoter Prediction (https://www.fruitfly.org/seq_tools/promoter.html), were amplified using the primers specific to the target regions (Table S4; hm1880-comp.-Fw and -Rv for *hm1880* complementation; hm2766-comp.-Fw and -Rv for *hm2766* complementation). The resulting DNA fragments and linearized pJRD-Cm^r^ were fused using the In-Fusion HD Cloning Kit (Takara Bio) according to the manufacturer’s instructions. Then, *E. coli* S17-1/λ*pir* harboring pJRD-P_native_-*hm1880* was conjugated with Δ*hm1880*, while that harboring pJRD-P_native_-*hm2766* was conjugated with Δ*hm2766* (Table S3). The transconjugants were selected on 1.5% LB agar plates containing Rif, Km, Cm, and Ura. The introduction of the constructed plasmids was verified by PCR with a primer pair of pJRD-check-Fw and -Rv (Table S4) and DNA sequencing. The empty vector-introduced strains Δ*hm1880*-pJRD and Δ*hm2766*-pJRD were used as vector controls.

### Quantification of EMV productivity and particle size distribution analysis of EMVs

To compare EMV productivity and particle diameters between the strains, EMV fractions were subjected to NTA using NanoSight NS300 (Malvern Panalytical Ltd., Malvern, United Kingdom). EMV solution diluted with DPBS was injected into the apparatus and analyzed with a camera level of 16 and a detection threshold of 5. EMV productivity and size distribution were analyzed based on these data using the NTA 3.4 Build 3.4.4 software (Malvern Panalytical Ltd.). EMV productivity was normalized to OD_600_ of the culture used for EMV isolation.

### SDS-PAGE analysis of secretory proteins

Each EMV fraction and PVF were mixed with 1/10 vol of 100% (wt/vol) trichloroacetic acid, incubated on ice for 15 min, and subsequently centrifuged at 20,300 × *g* for 15 min at 4°C to precipitate proteins. The pellets were washed with ice-cold acetone and re-precipitated by centrifugation at 20,300 × *g* for 5 min at 4°C. The dried protein pellet was suspended in SDS sample buffer (0.063 M Tris-HCl, 5% [vol/vol] 2-mercaptoethanol, 2% [wt/vol] SDS, 5% [wt/vol]) sucrose, and 0.005% [wt/vol] bromophenol blue) and incubated at 95°C for 5 min. The protein from 300 µL of culture was loaded onto a 12.5% SDS-PAGE gel. After electrophoresis, the gel was stained with CBB (Nacalai Tesque). To detect P49, western blot analysis was performed with an anti-P49 antibody according to the previously described method (20).

## SUPPLEMENTAL MATERIAL

Figure S1 jb.00497-24-s0001.pdf Violin plot of EMV hydrodynamic diameter.

Figure S2 jb.00497-24-s0002.pdf Transposon insertion sites of strain 26-F5 and strain 37-A11.

Figure S3 jb.00497-24-s0003.pdf Prediction of structure for HM1880 and HM1528.

Figure S4 jb.00497-24-s0004.pdf Growth of ΔPyrF^HM13^ under the static cultivation condition in 96-well plates.

Table S1 jb.00497-24-s0005.pdf Comparison of the relative EMV productivity evaluated by nFAAV5-NBD and NTA.

Table S2 jb.00497-24-s0006.pdf List of the domain hits searched by BLASTP.

Table S3 jb.00497-24-s0007.pdf Bacterial strains and plasmids used in this study.

Table S4 jb.00497-24-s0008.pdf Primers used in this study.

ASM does not own the copyrights to Supplemental Material that may be linked to, or accessed through, an article. The authors have granted ASM a non-exclusive, world-wide license to publish the Supplemental Material files. Please contact the corresponding author directly for reuse.
