# Supplementary material for: Rapid screening and identification of genes involved in bacterial extracellular membrane vesicle production using a curvature-sensing peptide
Source: J Bacteriol. 2025 Apr 4;207(5):e00497-24. doi: 10.1128/jb.00497-24 (PMC12096838; doi:10.1128/jb.00497-24)
Supplement: Figure S1 — Violin plot of EMV hydrodynamic diameter. [file jb.00497-24-s0001.pdf]

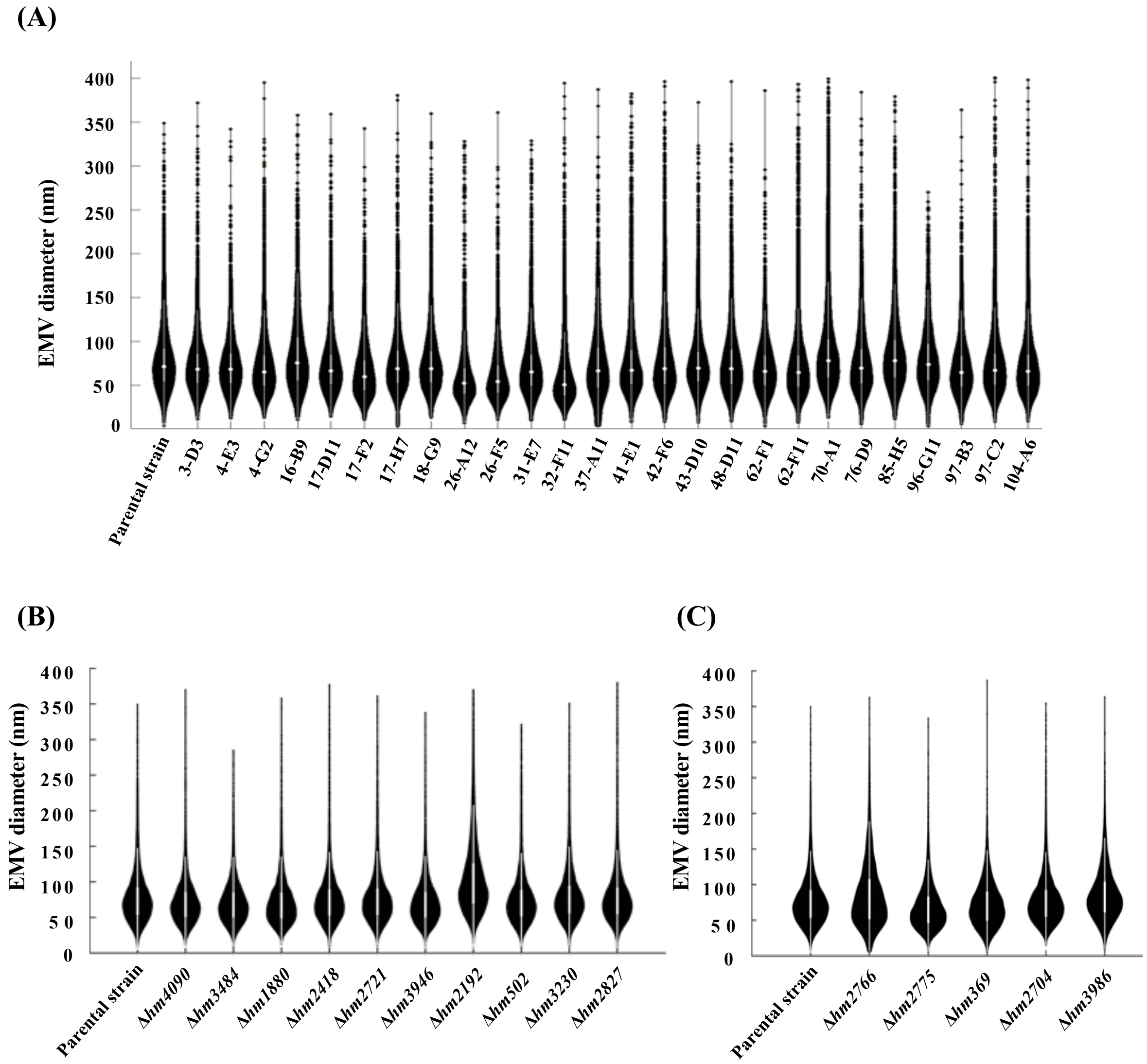

Fig. S1 Violin plot of EMV hydrodynamic diameter

The hydrodynamic diameter of EMVs was measured by NTA. (A) Tn mutants. (B) Mutants obtained by disruption of the genes identified from hyper-vesiculating Tn mutants. (C) Mutants obtained by disruption of the genes identified from hypo-vesiculating Tn mutants.
