# Supplementary material for: Rapid screening and identification of genes involved in bacterial extracellular membrane vesicle production using a curvature-sensing peptide
Source: J Bacteriol. 2025 Apr 4;207(5):e00497-24. doi: 10.1128/jb.00497-24 (PMC12096838; doi:10.1128/jb.00497-24)
Supplement: Figure S2 — Transposon insertion sites of strain 26-F5 and strain 37-A11. [file jb.00497-24-s0002.pdf]

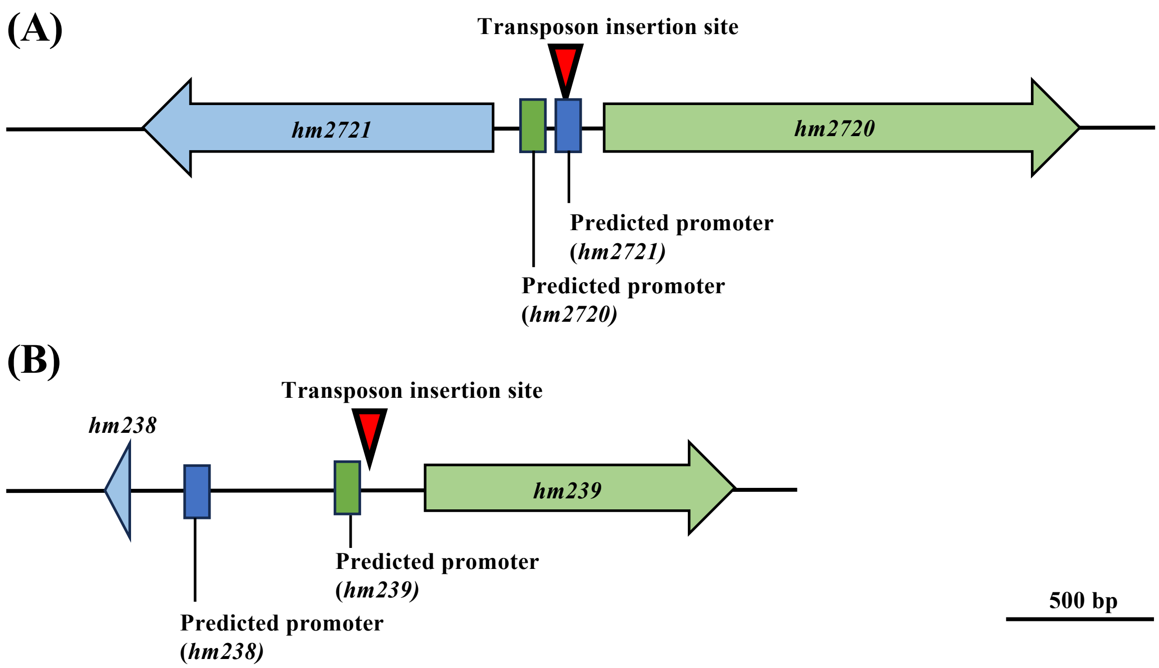

Fig. S2 Transposon insertion sites of strain 26-F5 and strain 37-A11
Transposon insertion sites were identified in strain 26-F5 (A) and strain 37-A11 (B). Promoter prediction was conducted using the neural network promoter prediction presented by Berkeley Drosophila Genome Project (https://www.fruitfly.org/seq_tools/promoter.html).
