# Supplementary material for: Rapid screening and identification of genes involved in bacterial extracellular membrane vesicle production using a curvature-sensing peptide
Source: J Bacteriol. 2025 Apr 4;207(5):e00497-24. doi: 10.1128/jb.00497-24 (PMC12096838; doi:10.1128/jb.00497-24)
Supplement: Figure S4 — Growth of ΔPyrFHM13 under the static cultivation condition in 96-well plates. [file jb.00497-24-s0004.pdf]

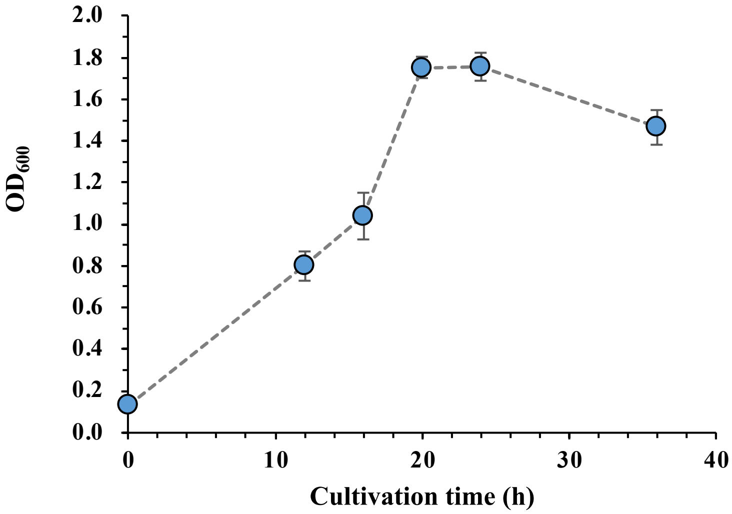


**Fig. S4 Growth of ΔPyrF$^{HM13}$ under the static cultivation condition in 96-well plates (Average +/- SD, n = 3)**

The growth of the parental strain under the static cultivation condition was examined. The strain grew to the stationary phase in 20−24 hours of cultivation. In the 1$^{st}$ selection of Tn mutants, the bacterium was grown for 24 hours to evaluate the EMV productivity of the mutants.
