# Supplementary material for: Rapid screening and identification of genes involved in bacterial extracellular membrane vesicle production using a curvature-sensing peptide
Source: J Bacteriol. 2025 Apr 4;207(5):e00497-24. doi: 10.1128/jb.00497-24 (PMC12096838; doi:10.1128/jb.00497-24)
Supplement: Table S2 — List of the domain hits searched by BLASTP. [file jb.00497-24-s0006.pdf]

Table S2 List of the domain hits searched by BLASTP

Protein	Length (a.a.)	Name	Accession	Description	Interval	E-value
HM502	529	PepSY_TM	pfam03929	PepSY-associated TM region	19–376	2.99E-60
		PiuB	COG3182	Uncharacterized iron-regulated membrane protein	19−381	2.71E-32
HM1880	219	COG3672	COG3672	Predicted transglutaminase-like cysteine proteinase	54−192	4.85E-36
		Peptidase_C93	pfam06035	Bacterial transglutaminase-like cysteine proteinase BTLCP	66−172	1.86E-11
HM2418	363	ComEC	COG2333	Metal-dependent hydrolase, beta-lactamase superfamily II	2−334	1.45E-11
		ComA-like_MBL-fold	cd07731	Competence protein ComA, ComEC and related proteins	4−270	2.37E-15
		Lactamase_B	smart00849	Metallo-beta-lactamase superfamily	31−98	1.44E-04
HM2192	502	Transglut_i_TM	pfam14400	Inactive transglutaminase fused to 7 transmembrane helices	25−184	7.40E-77
		7TM_transglut	pfam14402	7 transmembrane helices usually fused to an inactive transglutaminase	254−499	6.80E-133
HM2704	1615	Gdh2	COG2902	NAD-specific glutamate dehydrogenase	26−1610	0.00E+00
		Bac_GDH	pfam05088	Bacterial NAD-glutamate dehydrogenase	76−1605	0.00E+00
HM2766	808	PRK06464	PRK06464	phosphoenolpyruvate synthase	21−807	0.00E+00
		PEP_synth	TIGR01418	phosphoenolpyruvate synthase	23−806	0.00E+00
		PpsA	COG0574	Phosphoenolpyruvate synthase/pyruvate phosphate dikinase	23−806	0.00E+00
		PPDK_N	pfam01326	Pyruvate phosphate dikinase, PEP/pyruvate binding domain	35−369	8.46E-157
HM2775	281	YeaD	COG0676	D-hexose-6-phosphate mutarotase	6−272	2.90E-71
		D-hex-6-P-epi_like	cd09020	D-hexose-6-phosphate epimerase-like	16−278	7.86E-104
		Aldose_epim	pfam01263	Aldose 1-epimerase	19−278	2.02E-40
HM2827	1557	DUF6531	pfam20148	Domain of unknown function (DUF6531)	156−241	7.76E-20
		RHS_repeat	pfam05593	RHS proteins contain extended repeat regions	469−502	2.13E-03
		YD_repeat_2x	TIGR01643	YD repeat	490−531	4.14E-03
		RHS	pfam03527	RHS protein	1207−1242	6.06E-12
		Rhs_assc_core	TIGR03696	RHS repeat-associated core domain	1260−1325	1.00E-33
		HopBF1	cd20900	Type III secretion system (T3SS) effector HopBF1	1369−1527	9.42E-22
HM3484	468	gltD	PRK12810	Glutamate synthase subunit beta	1−467	0.00E+00
		GltD	COG0493	NADPH-dependent glutamate synthase beta chain or related oxidoreductase	23−467	0.00E+00
		Fer4_20	pfam14691	Dihydroprymidine dehydrogenase domain II, 4Fe-4S cluster	23−134	1.54E-56

HM3946	494	PRK05932	PRK05932	RNA polymerase factor sigma-54	1−494	0.00E+00
		RpoN	COG1508	DNA-directed RNA polymerase specialized sigma subunit, sigma54 homolog	1−494	1.04E-180
		rpoN_sigma	TIGR02395	RNA polymerase sigma-54 factor	10−492	1.13E-164
		Sigma54_DBD	pfam04552	Sigma-54, DNA binding domain	335−492	1.23E-91
HM3986	1236	PAS_4	pfam08448	PAS fold	465−573	3.40E-18
		PAS	COG2202	PAS domain	470−705	4.14E-21
		BaeS	COG0642	Signal transduction histidine kinase	670−953	9.23E-60
		PRK11107	PRK11107	hybrid sensory histidine kinase BarA; Provisional	711−1228	1.35E-133
		HATPase_EvgS-ArcB-TorS-like	cd16922	Histidine kinase-like ATPase domain of two-component sensor histidine kinases	838−945	2.30E-55
HM4090	716	Dcp	COG0339	Zn-dependent oligopeptidase	44−716	0.00E+00
		M3A_DCP	cd06456	Peptidase family M3, dipeptidyl carboxypeptidase (DCP)	70−714	0.00E+00
		Peptidase_M3	pfam01432	Peptidase family M3	268−714	1.62E-149

The E-value is the number of hits expected with a similar score by chance when searching the database with the amino acid sequence of each protein.
