# Supplementary material for: Rapid screening and identification of genes involved in bacterial extracellular membrane vesicle production using a curvature-sensing peptide
Source: J Bacteriol. 2025 Apr 4;207(5):e00497-24. doi: 10.1128/jb.00497-24 (PMC12096838; doi:10.1128/jb.00497-24)
Supplement: Table S3 — Bacterial strains and plasmids used in this study. [file jb.00497-24-s0007.pdf]

Table S3 Bacterial strains and plasmids used in this study

Strains and plasmids	Descriptions	References
**Strains**		
Escherichia coli S17-1/*λpir*	*E. coli* derivative, host for *pir*-dependent plasmids	(36)
ΔPyrF^{HM13}	Rifampicin resistant and *pyrF*-deletion mutant of *Shewanella vesiculosa* HM13	(35)
Δ*hm4090*	*hm4090*-disrupted mutant (*hm4090*:pKNOCK-Km) of ΔPyrF^{HM13}	This work
Δ*hm3484*	*hm3484*-disrupted mutant (*hm3484*:pKNOCK) of ΔPyrF^{HM13}	This work
Δ*hm1880*	*hm1880*-disrupted mutant (*hm1880*:pKNOCK) of ΔPyrF^{HM13}	This work
Δ*hm2418*	*hm2418*-disrupted mutant (*hm2418*:pKNOCK) of ΔPyrF^{HM13}	This work
Δ*hm2721*	*hm2721*-disrupted mutant (*hm2721*:pKNOCK) of ΔPyrF^{HM13}	This work
Δ*hm3946*	*hm3946*-disrupted mutant (*hm3946*:pKNOCK) of ΔPyrF^{HM13}	This work
Δ*hm2192*	*hm2192*-disrupted mutant (*hm2192*:pKNOCK) of ΔPyrF^{HM13}	This work
Δ*hm502*	*hm502*-disrupted mutant (*hm502*:pKNOCK) of ΔPyrF^{HM13}	This work
Δ*hm3230*	*hm3230*-disrupted mutant (*hm3230*:pKNOCK) of ΔPyrF^{HM13}	This work
Δ*hm2827*	*hm2827*-disrupted mutant (*hm2827*:pKNOCK) of ΔPyrF^{HM13}	This work
Δ*hm2766*	*hm2766*-disrupted mutant (*hm2766*:pKNOCK) of ΔPyrF^{HM13}	This work
Δ*hm2775*	*hm2775*-disrupted mutant (*hm2775*:pKNOCK) of ΔPyrF^{HM13}	This work
Δ*hm369*	*hm369*-disrupted mutant (*hm369*:pKNOCK) of ΔPyrF^{HM13}	This work
Δ*hm2704*	*hm2704*-disrupted mutant (*hm2704*:pKNOCK) of ΔPyrF^{HM13}	This work
Δ*hm3986*	*hm3986*-disrupted mutant (*hm3986*:pKNOCK) of ΔPyrF^{HM13}	This work
**Plasmids**		
pMiniHimar RB1	Transposon insertion. Containing *pir* dependent R6K ori plasmid. Km^r.	(37)
pKNOCK-Km	Gene knock-out. Containing *pir* dependent R6K ori plasmid. Km^r.	(24)
pJRD-Cm^r	Complementation. A broad-host-range vector. Cm^r.	(38)
pJRD-P$_{native}$-*hm1880*	Complementation. A pJRD-Cm^r derivative containing *hm1880*. Cm^r.	This work
pJRD-P$_{native}$-*hm2766*	Complementation. A pJRD-Cm^r derivative containing *hm2766*. Cm^r.	This work
