# Supplementary material for: Rapid screening and identification of genes involved in bacterial extracellular membrane vesicle production using a curvature-sensing peptide
Source: J Bacteriol. 2025 Apr 4;207(5):e00497-24. doi: 10.1128/jb.00497-24 (PMC12096838; doi:10.1128/jb.00497-24)
Supplement: Table S4 — Primers used in this study. [file jb.00497-24-s0008.pdf]

Table S4 Primers used in this study

Primers	Sequence (5'-3')	Target
pKNOCK-1	GGGCTGCAGGAATTCGATATCAAGC	pKNOCK-Km
pKNOCK-2	GGGGGATCCACTAGTTCTAGAGCG	As above
pKNOCK-check-FW	CCTCTCAAAGCAATTTTGAGTGACACAG	As above
pKNOCK-check-RV	TGCGTTTTCCCTTGTCCAGATAGC	As above
Himar1	GGGAATCATTTGAAGGTTGGTAC	pMiniHimar RB1
Himar615	TTCTTCTGAGCGGGACTCTGGG	As above
Single-Primer-PCR-1	TTCCAGTTTGAGATCTGG	As above
Single-Primer-PCR-2	AAGAATAGACCGAGATAGG	As above
Single-Primer-PCR-3	CCGAAATCGGCAAAATC	As above
Sigle-Primer-PCR-nested	CATTTAATACTAGCGACGCCATC	As above
hm4090-single-FW	ACTAGTGGATCCCCCTTTTTCAATCTGTCAGGGCTC	*hm4090*
hm4090-single-RV	GAATTCCTGCAGCCCGTTTCCCATAGCTGTTTACG	As above
hm4090-check-FW	CAACCGCTTATTTTTGAGTTTG	As above
hm3484-single-FW	ACTAGTGGATCCCCCTGTGCAAACAGTAGTGTATGACAAG	*hm3484*
hm3484-single-RV	GAATTCCTGCAGCCCCGCGAGCATTTTGTACTTCACG	As above
hm3484-check-FW	ATACATTACCGATACTGCCATATCACAAGG	As above
hm1880-single-FW	ACTAGTGGATCCCCCTTTAGCCAACCAGTTTGCTAAC	*hm1880*
hm1880-single-RV	GAATTCCTGCAGCCCCACGCACATACATCAAGCGC	As above
hm1880-check-FW	GCATGGATAAATATCGCCATTGGC	As above
hm2418-single-FW	ACTAGTGGATCCCCCCAGGGCTGACTGATAAGACAGG	*hm2418*
hm2418-single-RV	GAATTCCTGCAGCCCCCTGAATACCTGCATCCGCAG	As above
hm2418-check-FW	GGCCTGGAGGTGGTATTAAACG	As above
hm2721-single-FW	ACTAGTGGATCCCCCGTGGCTTGTTAGTGGCCAATG	*hm2721*
hm2721-single-RV	GAATTCCTGCAGCCCGCGCCAATGAGATAAACTCAACTG	As above
hm2721-check-FW	ATGCCATAGAACATATCCAAGGC	As above
hm3946-single-FW	ACTAGTGGATCCCCCGTGGCAAAAAAATCTCACGCC	*hm3946*
hm3946-single-RV	GAATTCCTGCAGCCCCTGGATTAAGGTGATTGCGTCACG	As above
hm3946-check-FW	CGACACAGCAGAATCCATGACC	As above
hm2192-single-FW	ACTAGTGGATCCCCCGCGACATCTATCGACATGATG	*hm2192*
hm2192-single-RV	GAATTCCTGCAGCCCAATAAGCTACCACCACCAGATAC	As above
hm2192-check-FW	TCGCCAACAACTGACATCATATG	As above
hm502-single-FW	ACTAGTGGATCCCCCCGAGCAAAAAAGGGCTGAATATCC	*hm502*
hm502-single-RV	GAATTCCTGCAGCCCATAACTGCCGCCAGGCAGATC	As above
hm502-check-FW	AGCCCATAGCTGGCTTGGAC	As above
hm3230-single-FW	ACTAGTGGATCCCCCTCCTGCCGGAGATGAGCTG	*hm3230*
hm3230-single-RV	GAATTCCTGCAGCCCTACGTCTTATGTCACCATGGAC	As above
hm3230-check-FW	AAAACCGCGACCAACATGGC	As above
hm2827-single-FW	ACTAGTGGATCCCCCAACAAGATAATCAAGACAAGCCTG	*hm2827*
hm2827-single-RV	GAATTCCTGCAGCCCTTGATAAAAGTTCTGAGTATTCAGTAGC	As above
hm2827-check-FW	GGACCGCGACGGTGAATTAC	As above
hm2766-single-FW	ACTAGTGGATCCCCCGCTAAGCAAGCCATGGTGATTG	*hm2766*
hm2766-single-RV	GAATTCCTGCAGCCCTGTTTACCCGAGAAAATTAAGCCAGT	As above
hm2766-check-FW	ATCGGCAGTAAGTTGATTCAGATGG	As above
hm2775-single-FW	ACTAGTGGATCCCCCGCGATCAATCTGTTGCGTTGC	*hm2775*
hm2775-single-RV	GAATTCCTGCAGCCCAACCACATCGTCGACTTGTTC	As above
hm2775-check-FW	GGCGCAAATAGATCAATTTACACCC	As above
hm369-single-FW	ACTAGTGGATCCCCCAAACTCAAGCAAACCAAAACCAG	*hm369*
hm369-single-RV	GAATTCCTGCAGCCCTTTTCCTGGTTTAATGGCCAGTTTC	As above
hm369-check-FW	TAGTGGGTAGCTCTTTTGCAGGC	As above
hm2704-single-FW	ACTAGTGGATCCCCCCTTCTTACGCTCTGCCAAGTCA	*hm2704*
hm2704-single-RV	GAATTCCTGCAGCCCTACGAGTCTGATCTTTACAGTCTTG	As above
hm2704-check-FW	TTGTCTTGCCACGCTCAAGC	As above
hm3986-single-FW	ACTAGTGGATCCCCCGCAGAAAGAATGCTGGGTTACCG	*hm3986*

hm3986-single-RV	GAATTCCTGCAGCCCCTGAGAGCGATATTCCTGCACC	As above
hm3986-check-FW	TCGACTCAATCCCTGAAGCG	As above
pJRD-Cm^r-1	TAGTATAGTCTATAGTCCGTGG	pJRD-Cm^r
pJRD-Cm^r-2	CGTAATCCATGGATCAAGAG	As above
pJRD-check-Fw	AGTTCTTCGAACAGTGCGCC	As above
pJRD-check-Rv	ATGAGAATTCCCCTGGATTTCACTG	As above
hm1880-comp.-Fw	GATCCATGGATTACGCTCTACTATTCGTTTTGTCATCGTTCC	*hm1880*
hm1880-comp.-Rv	CTATAGACTATACTACTATTCGCCATTTTCAATTCGATTGAC	As above
hm2766-comp.-Fw	GATCCATGGATTACGGGTGTTTTACCACAGCGAGATAC	*hm2766*
hm2766-comp.-Rv	CTATAGACTATACTATTAGCCATGAGCTTCAGCTAAATATAAC	As above

The sequences that anneal to pKNOCK-Km or pJRD-Cm^r are underlined.
